# Correlation between atrial cardiomyopathy and total burden of cerebral small vessel disease in patients with acute ischemic stroke

**DOI:** 10.3389/fneur.2026.1668267

**Published:** 2026-01-30

**Authors:** Qing He, Hui Zhang, Meiru Yi, Yongjun Jia, Meng Huang, Guoqin Huang, Daichao Ma

**Affiliations:** 1 The First School of Clinical Medicine, Shaanxi University of Chinese Medicine, Xianyang, China; 2 Department of Encephalopathy, Xi'an TCM Hospital of Encephalopathy, Xi'an, China; 3 Graduate School, China Academy of Chinese Medical Sciences, Beijing, China; 4 Department of Neurology, Affiliated Hospital of Shaanxi University of Chinese Medicine, Xianyang, China; 5 Department of Critical Care Medicine, Second Affiliated Hospital of Shaanxi University of Chinese Medicine, Xianyang, China; 6 Department of Radiology, Affiliated Hospital of Shaanxi University of Chinese Medicine, Xianyang, China; 7 Department of Ultrasound, Affiliated Hospital of Shaanxi University of Chinese Medicine, Xianyang, China

**Keywords:** atrial cardiomyopathy, cerebral small vessel disease, imaging markers, PTFV1, total Wardlaw scores

## Abstract

**Background:**

Atrial cardiomyopathy (ACM) and cerebral small vessel disease (CSVD) share common risk factors (e.g., hypertension, diabetes, dyslipidemia, aging) and pathophysiological mechanisms (e.g., inflammatory response, oxidative stress).

**Objective:**

This study aimed to investigate the relationship between ACM and the total CSVD burden in patients with acute ischemic stroke (AIS).

**Methods:**

We retrospectively enrolled eligible hospitalized AIS patients. Imaging markers were measured based on MRI data, including periventricular and deep white matter hyperintensities (WMH), enlarged perivascular spaces (EPVS), lacunes, cerebral microbleeds (CMBs), and brain atrophy. The total CSVD burden was calculated using the Wardlaw score. A P-wave terminal force in lead V1 (PTFV1) > 5,000 μV·ms was used to define atrial cardiomegaly (ACM), and a PTFV1 ≤ 5,000 μV·ms was adopted to define non-atrial cardiomegaly (NACM). Univariate and multivariate ordinal logistic regression analyses estimated the correlation between ACM and total CSVD burden.

**Results:**

Among 323 enrolled patients (mean age 67.67 years, 63.7% male), 83 were classified as ACM. Patients with ACM had significantly higher total Wardlaw scores (OR = 1.79, 95% CI = 1.07–3.02, *p* = 0.026). Age, hypertension, and NIHSS score were also independent risk factors for increased total burden [OR = 1.05 (95% CI = 1.02–1.07, *p* < 0.001), 2.09 (95% CI = 1.29–3.42, *p* = 0.003), and 1.13 (95% CI = 1.03–1.24, *p* = 0.009), respectively].

**Conclusion:**

This study found a suggestive association between ACM and total CSVD burden in AIS patients, underscoring the importance of assessing ACM for evaluating CSVD risk factors in this patient population.

## 1 Introduction

Atrial cardiomyopathy (ACM) refers to a disease characterized by alterations in atrial morphology, structure, function, or electrophysiology, accompanied by relevant clinical symptoms (1). Specifically, ACM can occur secondary to atrial fibrillation or manifest as primary structural/functional changes independent of atrial fibrillation (2). Its key features include reduced blood flow velocity and atrial fibrosis, both of which can promote thrombogenesis and thromboembolism independently of atrial fibrillation (2). Notably, impaired left atrial mechanical function has been associated with ischemic cerebrovascular events, even in the absence of incident atrial fibrillation (3). Moreover, ACM may progress to atrial fibrillation, thereby further elevating stroke risk (4, 5). In addition, atrial myopathy compromises left atrial reservoir, conduit, and contractile functions, reducing emptying while increasing stiffness; this, in turn, elevates left atrial pressure and potentially leads to pulmonary venous hypertension (2).

Cerebrovascular damage resulting from atrial structural and functional abnormalities has been well established in large vessels, but its relationship with CSVD deserves attention. As a syndrome, CSVD involves pathological changes in small intracerebral arteries, arterioles, capillaries, venules, and small veins, presenting with clinical, radiological, and neuropathological manifestations (6). It contributes to cognitive decline, gait disturbances, urinary dysfunction, and significantly impairs quality of life (7). The interconnection between ACM and CSVD may operate through several mechanisms: (1) They share common risk factors such as aging, diabetes, and hypertension (1, 8); (2) Systemic inflammation and oxidative stress drive the progression of both ACM (2, 9) and CSVD (10, 11); (3) There may be bidirectional causality: CSVD lesions could damage sympathetic pathways regulating cardiovascular function in subcortical and brainstem regions, thereby affecting left atrial function via the brain-heart axis (12). Conversely, ACM can lead to the formation of intra-atrial thrombi (regardless of atrial fibrillation presence), which may act as embolic sources for CSVD-related lacunar infarcts (13, 14).

A P-wave terminal force in ECG lead V1 (PTFV1) ≥ 5,000 μV·ms is widely accepted diagnostic criteria for ACM (15). An abnormal PTFV1 reflects left atrial dysfunction (16) and serves as a marker of electrophysiological remodeling (17). Studies have linked abnormal PTFV1 to cryptogenic or cardioembolic stroke (18–20), leukoaraiosis (21), and subcortical infarction (19). However, the association between ACM and total CSVD burden in patients with acute ischemic stroke (AIS) remains unclear. Therefore, this study aimed to investigate the correlation between ACM and total CSVD burden in patients diagnosed with AIS.

## 2 Materials and methods

### 2.1 Study design and participants

This retrospective observational study continuously enrolled AIS patients admitted to the Affiliated Hospital of Shaanxi University of Chinese Medicine between February 2022 and December 2023. AIS was defined as sudden neurological dysfunction due to focal cerebral ischemia, with symptoms lasting >24 h or neuroimaging evidence of acute infarction (22). Inclusion criteria: (1) Age ≥18 years; (2) Imaging-confirmed acute infarction; (3) Complete clinical data, including demographics, cardiovascular risk factors, medical and medication history, echocardiography, and stroke causative mechanisms. Exclusion criteria: (1) Intracerebral hemorrhage or venous infarction; (2) Persistent atrial fibrillation during hospitalization (a condition precluding reliable P-wave detection); (3) History of carotid stenting; (4) Brain tumor, malignancy, head trauma, severe systemic disease (e.g., heart failure, renal failure/dialysis, respiratory failure), liver failure, substance/alcohol abuse, psychiatric disorders, or other neurological diseases (e.g., multiple sclerosis, encephalopathy, chorea, Parkinson’s); (5) Non-vascular white matter lesions (e.g., autoimmune, metabolic, toxic, infectious, sarcoidosis, radiation-induced); (6) MRI contraindications.

### 2.2 Ethics approval and consent

The study was approved by the Ethics Committee of Shaanxi University of Chinese Medicine Affiliated Hospital [Approval No. 2023(77)] and adhered to the Declaration of Helsinki. Written informed consent was obtained from all participants.

### 2.3 Baseline data collection

Comprehensive baseline data were collected: Demographics (sex, age, educational years, height, weight); Cardiovascular risk factors (systolic/diastolic blood pressure (SBP/DBP), smoking, alcohol use, heart rate, fasting plasma glucose (FPG), HbA1c%, total cholesterol (TC), low density lipoprotein cholesterol (LDL), triglycerides (TG)); Medical history (hypertension, diabetes, atrial fibrillation); Medication history (antihypertensives, antiplatelets, anticoagulants) within 3 months; Inflammatory markers [fibrinogen (Fbg), homocysteine (HCY)]. The presumed stroke causative mechanism was assessed based on the Trial of ORG 10172 in Acute Stroke Treatment (TOAST) classification. Data were extracted from medical records via dual verification.

### 2.4 MRI acquisition and analysis

Brain MRI scans (Siemens 1.5 T/3.0 T MAGNETOM SKYRA) were retrieved from the hospital’s imaging system. Sequences included DWI, T1WI, T2WI, FLAIR, and SWI. Measurements followed Standards for Reporting Vascular Changes on Neuroimaging (STRIVE) (23): (1) Lacunes: Defined as round or ovoid, subcortical CSF-signal lesions (3–15 mm diameter) on T1/T2/FLAIR. Number counted visually. (2) White Matter Hyperintensities (WMH): Abnormal hyperintense signals in white matter on T2/FLAIR. Severity was quantified using the Fazekas scale (24) for periventricular WMH (PV-WMH) and deep WMH (D-WMH), summed for a total WMH score (0–6). WMH volume (mm^3^) was measured using 3D Slicer (v5.0.3) by semi-automated thresholding and manual correction. WMH severity was expressed as a percentage of intracranial volume (ICV), measured similarly. (3) Enlarged Perivascular Spaces (EPVS): Defined as linear (parallel to vessels) or round/ovoid (diameter <3 mm, CSF-signal) hyperintensities on T2WI. Centrum semiovale (CSO) and basal ganglia (BG) EPVS counts were visually graded (25): Grade 0 (none), 1 (≤10), 2 (11–20), 3 (21–40), 4 (>40). (4) Cerebral Microbleeds (CMBs): Defined as small, round/ovoid, homogeneous, signal-void lesions (2–5 mm, max 10 mm) on SWI. Burden was graded (26): 0 (none), 1 (1–5), 2 (6–15), 3 (>15). (5) Brain Atrophy: Assessed by reduced brain volume, ventricular enlargement, and sulcal widening on MRI. Graded using the Global Cortical Atrophy (GCA) scale (27): 0 (none), 1 (mild), 2 (moderate), 3 (severe). Two trained raters, blinded to clinical data, assessed all CSVD markers. Disagreements were resolved by a senior physician. (6) Total CSVD Burden: Wardlaw score (0–4) (25): 1 point each for: D-WMH (Fazekas scale 2 or 3) or irregular PV-WMH (Fazekas score 3), presence of≥1 lacunes, presence of≥1 CMB, moderate/severe BG-EPVS (*N* > 10). The markers on MRI images were independently assessed by Q. H. and M. Y. This assessment was conducted while the raters were blinded to the clinical data of the participants. Any discrepancies between their assessments were resolved by Y. J. The kappa coefficients and corresponding *p*-values of CSVD markers on brain MRI between the two raters are listed as follows: Lacunes (0.724, *p* < 0.01), PV-WMH (0.868, *p* < 0.01), D-WMH (0.863, *p* < 0.01), tolat WMH (0.826, *p* < 0.01), EPVS grade (0.859, *p* < 0.01), CSO-EPVS (0.559, *p* < 0.01), BG-EPVS (0.630, *p* < 0.01), CMBs-grade (0.888, *p* < 0.01), CBM numbers (0.689, *p* < 0.01), and Atrophy grade (0.839, *p* < 0.01).

### 2.5 Echocardiography

Transthoracic echocardiography (Philips EPIQ5) assessed cardiac structure/function during hospitalization. Parameters recorded: Left atrial diameter index (LADI); Measurements followed American Society of Echocardiography guidelines by an experienced sonographer blinded to other data. LADI were derived by LAD/BSA. Body surface area (BSA, m^2^) was calculated (28): Male S = 0.0057*height (cm) + 0.0121*weight (kg) + 0.0882; Female S = 0.0073*height (cm) + 0.0127*weight (kg) - 0.2106.

### 2.6 Electrocardiography

PTFV1 was calculated as the product of depth (μV) and duration (ms) of the terminal portion of the P-wave in V1. The 12-lead routine ECG was used, and the ECGs of all patients were collected in the resting state on the first day after admission. Paper ECGs were scanned, magnified 10 in Digimizer software, and three non-consecutive inverted P-waves in V1 were measured and averaged. Measurements were blinded. All ECGs were calibrated to standard settings (10 mm/mV, 25 mm/s). A P-wave terminal force in lead V1 (PTFV1) > 5,000 μV·ms was used to define atrial cardiomegaly (ACM), and a PTFV1 ≤ 5,000 μV·ms was adopted to define non-atrial cardiomegaly (NACM). Patients with unstable baselines or absent P-waves due to atrial fibrillation were excluded. Two measurers performed assessments; discrepancies were resolved by a senior cardiologist.

### 2.7 Statistical analysis

Continuous variables are presented as mean±SD or median (IQR); categorical variables as frequency (%). Group comparisons used Student’s t-test, Mann–Whitney U test, Chi-square test, or Fisher’s exact test. Baseline and echocardiographic characteristics were compared between ACM (PTFV1 > 5,000 μV·ms) and non-ACM (NACM, PTFV1 ≤ 5,000 μV·ms) groups. Ordinal logistic regression assessed associations between echocardiographic parameters and total Wardlaw scores. Univariate and multivariate ordinal logistic regression identified independent risk factors for higher CSVD burden (total Wardlaw score as dependent variable; clinical/laboratory parameters as independent variables). Variables with *p* value ≤ 0.05 in univariate analysis were entered multivariate analysis. The standardized variance inflation factor (SVIF) < 1.5 was adopted as the critical threshold for determining the absence of multicollinearity among variables. It is calculated as GVIF^(1/(2*df)), where GVIF denotes the generalized variance inflation factor and df denotes degrees of freedom. Multivariate ordinal logistic regression was constructed based on the total Wardlaw score, followed by overall significance testing and goodness-of-fit evaluation: (1) The Brant test was employed to validate the proportional odds assumption (POA), assessing whether the effects of independent variables on each category of the dependent variable were consistent; (2) The likelihood ratio test (LRT) was used to compare the full model (incorporating 8 independent variables including ACM and hypertension) with the null model (intercept-only), to verify the joint explanatory power of the independent variables for the ordered categories of the total Wardlaw score; (3) Pseudo R^2^ and the Hosmer-Lemeshow (HL) test were utilized to evaluate the model’s goodness-of-fit to the data. The Cochran-Armitage trend test was used to assess the linear trend in the distribution of total Wardlaw scores across ACM and NACM groups. Statistical significance was set at *p* value < 0.05. Analyses used R software (v4.0.1).

## 3 Results

### 3.1 Clinical characteristics

Of 426 screened AIS patients, 103 were excluded (32 incomplete echo data, 15 unreadable ECG, 11 > 7 days post-stroke, 45 MRI artifacts/missing sequences), leaving 323 patients (240 NACM, 83 ACM). Baseline characteristics are shown in Table 1. No significant differences existed in age, sex, weight, height, BMI, education, hypertension/diabetes/atrial fibrillation history, medication use (antiplatelets, antihypertensives), or lab values (FPG, HbA1c, TG, TC, HDL, LDL, HCY, Fbg). Statistically significant differences were found only in heart rate, SBP, DBP, and LADI (higher in ACM group).

**Table 1:** Demographic and clinical characteristics.

Characteristic	NACM (*n* = 240)	ACM (*n* = 83)	*P*-value
Demographics			
Age, median (IQR)	69.00 [60.00, 76.00]	70.00 [59.00, 78.50]	0.445
Male, *n* (%)	149 (62.1)	57 (68.7)	0.345
Weight, median (IQR), kg	66.25 [60.00, 73.00]	70.00 [61.50, 75.50]	0.088
Height, median (IQR), cm	167.00 [161.75, 171.25]	168.00 [162.00, 172.50]	0.307
BSA, median (IQR), m^2^	1.85 [1.74, 1.94]	1.89 [1.78, 1.97]	0.092
Education, median (IQR), yrs	11.00 [8.00, 11.00]	11.00 [8.00, 11.00]	0.648
Heart rate, median (IQR), bpm	74.50 [69.00, 83.00]	78.00 [70.00, 89.50]	**0.021**
SBP, median (IQR), mmHg	140.00 [127.00,160.00]	150.00 [135.00,166.50]	**0.004**
DBP, median (IQR), mmHg	84.00 [76.00, 93.00]	88.00 [79.50,101.00]	**0.012**
Risk factors, *n* (%)			
Active smoking	118 (49.2)	41 (49.4)	1.000
Alcohol consumption	116 (48.3)	34 (41.0)	0.302
Hypertension	158 (65.8)	56 (67.5)	0.891
Diabetes	65 (27.1)	27 (32.5)	0.420
Atrial fibrillation	6 (2.5)	3 (3.6)	0.885
TOAST subtypes			
Large artery atherosclerosis	25 (10.4)	12 (14.5)	0.483
Undetermined etiology	3 (1.2)	2 (2.4)	
Other determined etiology	2 (0.8)	1 (1.2)	
Small-vessel occlusion	207 (86.2)	66 (79.5)	
Cardioembolism	3 (1.2)	2 (2.4)	
Medication, *n* (%)			
Antiplatelet	92 (38.3)	39 (47.0)	0.434
Antihypertensive	125 (52.1)	50 (60.2)	0.247
Laboratory data, median (IQR)			
FPG, mmol/L	5.60 [4.90, 7.22]	5.70 [5.24, 8.00]	0.353
HbA1c, %	5.70 [5.30, 6.70]	5.80 [5.40, 7.30]	0.153
Triglycerides, mmol/L	1.40 [0.98, 1.97]	1.43 [1.04, 1.90]	0.616
TC, mmol/L	4.24 [3.48, 5.16]	4.41 [3.48, 5.00]	0.989
HDL, mmol/L	1.10 [0.95, 1.29]	1.13 [0.94, 1.26]	0.811
LDL, mmol/L	2.42 [1.83, 3.09]	2.45 [1.96, 3.09]	0.708
HCY, μmol/L	18.80 [14.80, 26.05]	18.70 [14.90, 24.80]	0.949
Fbg, g/L	2.89 [2.39, 3.56]	2.92 [2.45, 3.48]	0.921
Echocardiographic parameters			
LADI, cm/m^2^	1.73 [1.57, 1.89]	1.85 [1.67, 2.00]	**0.001**

BSA, body surface area; DBP, diastolic blood pressure; Fbg, Fibrinogen; FPG, fasting plasma glucose; HbA1c, hemoglobin A1c; HDL, high-density lipoprotein; HCY, homocysteine; LDL, low-density lipoprotein; PTFV1, P-wave terminal Force in V1 lead; SBP, systolic blood pressure; TC, total cholesterol; LADI, left atrial diameter index.

### 3.2 Association between ACM and total CSVD burden

Univariate ordinal logistic regression (Table 2) identified age, hypertension, NIHSS scores, TOAST Subtypes, ACM, triglycerides, HDL, and Fbg as significant risk factors for higher total Wardlaw scores.

**Table 2:** Univariate ordinal regression analysis for predictors of total Wardlaw score.

Variables	OR (95% CI)	*P*-value
Female sex	1.25 (0.81–1.94),	0.315
Age, years	1.05 (1.03–1.07),	**<0.001**
Weight, kg	1.00 (0.98–1.02),	0.808
Height, cm	0.98 (0.95–1.01),	0.270
Smoking	1.02 (0.67–1.56),	0.920
Alcohol use	0.79 (0.52–1.21),	0.281
BSA, m^2^	0.73 (0.19–2.72),	0.638
Education, yrs	0.99 (0.93–1.05),	0.684
Heart rate, bpm	1.00 (0.99–1.02),	0.681
Hypertension	1.99 (1.27–3.15),	**0.003**
SBP, mmHg	1.01 (1.00–1.02),	0.111
DBP, mmHg	1.01 (0.99–1.02),	0.375
Antihypertensive	1.51 (0.99–2.31),	0.057
Diabetes	1.42 (0.89–2.27),	0.146
Atrial fibrillation	0.83 (0.24–2.88),	0.763
NIHSS scores	1.14 (1.06–1.23),	**<0.001**
ACM	1.74 (1.07–2.86),	**0.026**
TOAST subtypes (Large artery atherosclerosis as reference)		
Undetermined etiology	0.42 (0.06–2.52),	**0.019**
Other determined etiology	0.10 (0.01–1.02)	
Small-vessel occlusion	0.83 (0.42–1.63)	
Cardioembolism	12.59 (1.68–112.03)	
Laboratory data		
FPG, mmol/L	1.05 (0.97–1.14)	0.236
HbA1c, %	1.05 (0.97–1.14)	0.396
Triglycerides, mmol/L	0.86 (0.75–0.98)	**0.020**
TC, mmol/L	0.93 (0.77–1.12)	0.421
HDL, mmol/L	2.11 (1.00–4.50)	**0.050**
LDL, mmol/L	0.88 (0.69–1.12)	0.314
HCY, μmol/L	1.01 (1.00–1.02)	0.081
Fbg, g/L	1.53 (1.18–2.00)	**0.001**
LADI	2.09 (0.97–4.71)	0.058

Eight variables screened by univariate ordinal regression analysis were entered into multivariate ordinal logistic regression analysis (Table 3). Multicollinearity analysis was conducted for the eight independent variables included in the ordinal logistic regression model. The results revealed that the SVIF were all less than 1.5, indicating no multicollinearity among all variables. The results of model validation and testing are as follows: (1) Validation of the proportional odds assumption: The Brant test yielded an omnibus chi-square value of 15.6 (*p* = 0.84), indicating that the model satisfied the proportional odds assumption. (2) Overall significance of the regression equation: The LRT results showed that, compared with the null model, the full model produced a chi-square value of 66.93 (*P* < 0.001). This demonstrates that the independent variables, when considered jointly, have significant explanatory power for the ordered categories of the total Wardlaw score, and the regression equation is statistically significant overall. (3) Model goodness-of-fit: The Nagelkerke pseudo-R^2^ of the model was 0.218, suggesting a moderate level of explanatory power. The HL test yielded a chi-square value of 9.28 (*p* = 0.319), confirming that the model exhibits a good fit.

**Table 3:** Multivariate ordinal regression analysis for predictors of total Wardlaw score.

Variables	OR (95% CI)	*P*-value
Age, years	1.05 (1.02–1.07)	**<0.001**
Hypertension	2.09 (1.29–3.42)	**0.003**
NIHSS scores	1.13 (1.03–1.24)	**0.009**
ACM	1.79 (1.07–3.02)	**0.026**
TOAST Subtypes (Large artery atherosclerosis as reference)		
Undetermined etiology	2.86 (0.37–20.29)	0.136
Other determined etiology	0.40 (0.03–4.64)	
Small-vessel occlusion	1.36 (0.60–3.13)	
Cardioembolism	10.51 (1.40–113.46)	
Triglycerides, mmol/L	0.91 (0.79–1.05)	0.199
HDL, mmol/L	1.01 (0.44–2.34)	0.978
Fbg, g/L	1.21 (0.91–1.61)	0.191

Multivariate analysis (adjusting for TOAST Subtypes, TG, HDL, and Fbg) confirmed ACM as an independent predictor of higher total Wardlaw scores (OR = 1.79, 95% CI = 1.07–3.02, *p* = 0.026). This indicates that, after adjusting for confounding factors, the cumulative odds of having a more severe small vessel disease burden (i.e., being in a higher Wardlaw score category) are 1.79 times higher for patients with ACM compared to those with NACM. In addition, age (OR = 1.05, 95% CI = 1.02–1.07, *p* < 0.001), hypertension (OR = 2.09, 95% CI = 1.29–3.42, *p* = 0.003), and NIHSS score (OR = 1.13, 95% CI = 1.03–1.24, *p* = 0.009) were also independent risk factors. This indicates that for each 1-year increase in age, the adjusted cumulative odds of being in a higher Wardlaw score category increase by a factor of 1.05. The cumulative odds of a higher Wardlaw score are 2.09 times greater in patients with hypertension than in those without. Each one-point increase in NIHSS score is associated with a 1.13-fold increase in the cumulative odds of a higher Wardlaw score.

**Figure 1:**
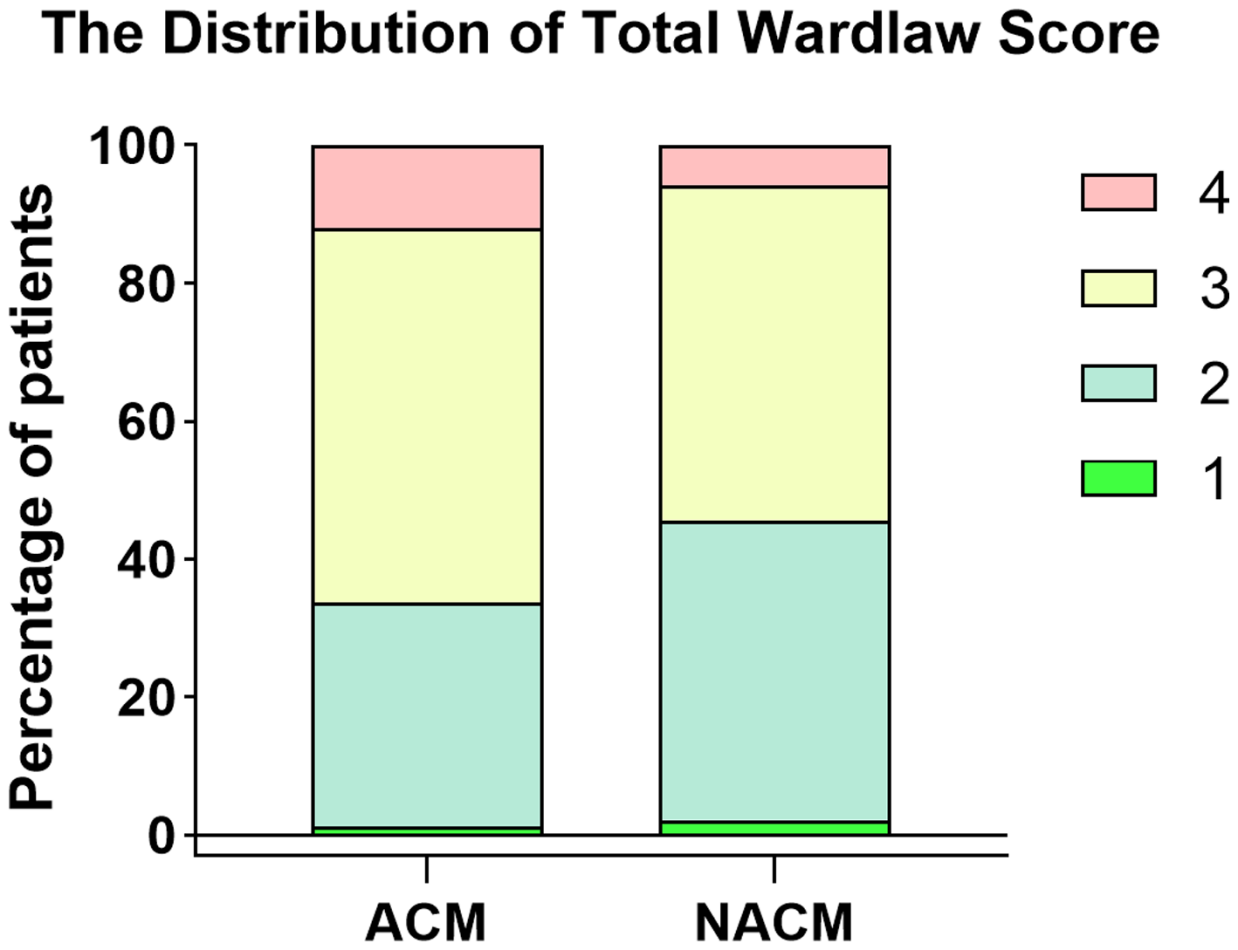
The distribution of total Wardlaw score. Bar chart showing higher percentages of scores 3 and 4 in ACM group compared to NACM group.

The ACM group had significantly higher proportions of patients with scores of 3 and 4 compared to NACM (*p* value = 0.023 for ordinal difference). Figure 1 illustrates the distribution of total Wardlaw score.

## 4 Discussion

The total CSVD score, which offers a comprehensive assessment of the overall cerebral burden imposed by CSVD, has emerged as a valuable instrument for both clinical trials and routine clinical practice (25, 29, 30). This study aimed to investigate the correlation between ACM and total CSVD burden in patients diagnosed with AIS. The main result indicated that ACM was significantly associated with CSVD burden, underscoring the importance of assessing left atrial abnormalities for evaluating CSVD risk factors in patients with AIS.

Our findings also showed that age and hypertension were independent predictors of higher total CSVD burden, and this aligns with the established roles of age and hypertension as key CSVD risk factors (8). Furthermore, the association between a higher NIHSS score (indicating stroke severity) and a heavier CSVD burden supports findings by Arba and Zerna (31, 32) that link CSVD burden to ischemic stroke occurrence, progression, and poor outcomes. Clinically, therefore, evaluating left atrial abnormalities in patients with AIS and intervening on shared risk factors may help improve prognosis of patients.

The association between ACM and CSVD may operate through several underlying mechanisms: (1) Shared traditional risk factors, including hypertension, diabetes, dyslipidemia, and aging (1, 8). (2) Co-promotion by systemic inflammation and oxidative stress (2, 9–11). (3) Potential disruption of the brain-heart axis: CSVD lesions may impair central sympathetic pathways that regulate cardiovascular function, thereby affecting left atrial function (12). Our data show that patients in the ACM group had a higher heart rate compared with those in the NACM group, which tentatively raises the possibility of potential increased sympathetic nerve tone in the ACM group. (4) ACM can lead to the formation of intra-atrial microthrombi (regardless of atrial fibrillation presence), which may act as embolic sources for CSVD-related lacunar infarcts to a certain extent (13, 14, 33). Taken together, these underlying mechanisms collectively underscore the complex interplay between ACM and CSVD, providing a multifaceted framework for understanding their association.

## 5 Limitation

While our study affords meaningful insights into the association between ACM and total CSVD burden, it is important to acknowledge several inherent limitations. Firstly, it uses a single-center, retrospective design with a moderate sample size, which may introduce bias. Secondly, the diagnosis of ACM relied solely on a PTFV1. NT-proBNP was not measured routinely, and only one patient met the LADI criterion in our data. This suggests that PTFV1 has higher sensitivity, consistent with the findings of Didier et al. (34), in which 45.19% of patients met the PTFV1 criteria compared with 3.84% for LADI. Furthermore, PTFV1 was measured from ECGs. Patients with persistent atrial fibrillation were excluded from the study based on the exclusion criteria, as PTFV1 cannot be accurately measured in this subgroup. The ACM severity in this subgroup may be more pronounced, it may lead to the underestimation of our results. Thus, future prospective studies employing comprehensive diagnostic criteria (including PTFV1, NT-proBNP, and echocardiographic parameters) in larger cohorts are necessary; finally, to investigate the relationship between atrial functional alterations and total CSVD burden, patients with heart failure were excluded from the present study. Emerging evidence has demonstrated a significant correlation between heart failure and CSVD, particularly with cerebral microbleeds (35). This may consequently limit the generalizability of the study’s findings.

## 6 Conclusion

In conclusion, this study the present study provides evidence of a suggestive association between ACM and total CSVD burden in patients with AIS. Specifically, ACM, defined by abnormal PTFV1, closely correlates with CSVD severity. This association is likely underpinned by shared risk factors, common pathophysiological drivers, and potential bidirectional interactions, including autonomic dysfunction via the brain-heart axis and microembolic mechanisms. These findings highlight the clinical relevance of assessing left atrial functional abnormalities for evaluating CSVD risk. Assessing ACM for evaluating CSVD risk factors in patients with AIS and implementing targeted interventions for their shared risk factors, such as hypertension control, could potentially improve patients’ prognosis and quality of life. Exploratory studies investigating anticoagulant therapy in ACM patients without atrial fibrillation, with a focus on observing the preventive effect of this therapy on the progression of lacunar lesions, would be of great significance for clarifying the pathogenesis of lacunar lesions derived from embolic sources in non-atrial fibrillation related ACM. In the future, it may be feasible to establish a risk stratification system for the management of CSVD patients based on whether they have comorbid ACM. However, given the limitations of the current single-center retrospective design and reliance on PTFV1 for ACM diagnosis, future large-scale prospective studies incorporating comprehensive diagnostic criteria are warranted.

## Data Availability

The raw data supporting the conclusions of this article will be made available by the authors, without undue reservation.
